# In Vitro Effects of Photobiomodulation with 660 Nm Laser and Vitamin D on Osteoblastic Differentiation of Human Periodontal Ligament Stem Cells

**DOI:** 10.31661/gmj.v13i.3312

**Published:** 2024-05-05

**Authors:** Hormoz Dehghani Soltani, Maryam Tehranchi, Ferial Taleghani, Sogol Saberi, Mahshid Hodjat

**Affiliations:** ^1^ Department of Periodontics, Faculty of Dentistry, Shahed University, Tehran, Iran; ^2^ Laser Research Center of Dentistry, Dentistry Research Institute, Tehran University of Medical Sciences, Tehran, Iran; ^3^ Dentistry Research Institute, Tehran University of Medical Sciences, Tehran, Iran GMJ.2024;13:e3312 www.gmj.ir

**Keywords:** Photobiomodulation Therapy, Mesenchymal Stem Cells, Cell Differentiations, Cellular Engineering; Vitamin D

## Abstract

Background: Mesenchymal stem cells (MSCs) can be found inside the human periodontal ligament. Application of vitamin D and photobiomodulation for regulation of the proliferation of MSCs and bone differentiation have been recently considered in cell engineering. This study is performed to evaluate the effects of photobiomodulation with 660 nm laser exposure and vitamin D on human periodontal ligament stem cells (HPDLSCs) and their osteoblastic differentiation properties. Materials and Methods: This study, was an in vitro experimental study performed on HPDLSCs in six groups of (I) control cells in the culture medium with no intervention, (II) addition of 10-7 mol vitamin D to the medium, (III) 660 nm diode laser exposure in 3 J/cm2 density of energy, (IV) 660 nm diode laser exposure in 3 J/cm2 density of energy + addition of 10-7 mol vitamin D to the medium, (V) 660 nm diode laser exposure in 5 J/cm2 density of energy, and (VI) 660 nm diode laser exposure in 5 J/cm2 density of energy + addition of 10-7 mol vitamin D to the medium. after 24 hours of the last exposure, cell viability had been assessed by methyl thiazolyl tetrazolium assay. The expression of Runt-related transcription factor 2 (RUNX2), osteopontin (OPN), alkaline phosphatase (ALP), and osteocalcin (OCN) genes was also assessed by reverse transcription-polymerase chain reaction, then Alizarin red staining was used to assess calcification. Results: Combined use of 660 nm laser with 3 and 5 J/cm2 density of energy and 10-7 mol vitamin D significantly increased cell viability, osteoblastic differentiation by upregulation of RUNX2, ALP, OPN, and OCN, and calcification (P0.05). Conclusion: The results showed that combined use of vitamin D3 and irradiation of 660 nm laser with 3 J/cm2 and particularly 5 J/cm2 energy density increased the viability of HPDLSCs and enhanced their osteoblastic differentiation.

## Introduction

**Table:** Table1. Nucleotide sequence of real-time primers

Gene	Sequence
OCN	F-TCACACTCCTCGCCCTATTG
	R-GCTCCCAGCCATTGATACAG
OPN	F-TCCAACGAAAGCCATGACCA
	R-GCAGGTCCGTGGGAAAATCA
GAPDH	F-CACATGGCCTCCAAGGAGTAA
	R-TGAGGGTCTCTCTCTTCCTCTTG
ALP	F-GCTGTAAGGACATCGCCTACCA
	R-CCTGGCTTTCTCGTCACTCTCA
RUNX2	F-GGAGTGGACGAGGCAAGAGTT
	R-GGTTCCCGAGGTCCATCTACT

OCN; OPN; GAPDH: Glyceraldehyde 3-phosphate dehydrogenase; ALP; RUNX2

Tissue engineering has become a new way to manage tissue defects, especially critical-size defects. In this method, stem cells are used to produce differentiated functional cells and regenerate a functional tissue, instead of merely filling the defect with graft material [1]. In tissue engineering, a combination of dental material science, cell biology, and tissue engineering is used for the reconstruction and regeneration of the lost tissue [2]. Four major challenges in tissue engineering need to be optimized, namely sources of cells, biomaterials, angiogenesis, and delivery systems of drugs [2]. Mesenchymal stem cells (MSCs) are among the best sources of cells for tissue engineering of the bone [1]. Human periodontal ligament stem cells (HPDLSCs) are a special source of MSCs used for this purpose [3]. Seo et al. [4] confirmed the potential of stem cells isolated from the PDL adjacent to the middle third of the root surface for tissue engineering to regenerate new functional periodontal tissue [5].

The differentiation steps of stem cells to osteoblasts include differentiation into immature osteoprogenitor cells, mature osteoprogenitor cells, pre-osteoblasts, mature osteoblasts, and osteocytes. The presence of specific proteins in the culture medium such as bone morphogenetic proteins enhances cell differentiation and formation of new osteoblasts and subsequent calcification [6].

On the other hand, the direct anabolic effect of vitamin D on osteoblasts has been confirmed and also it can indirectly enhance the proliferation of bone cells by increasing the calcium uptake [7]. Moreover, it has been well confirmed that the active form of vitamin D, i.e., 1,25-(OH)2D3 can induce the differentiation of MSCs to osteoblasts in vitro, especially by binding to the vitamin D receptor in the cell nucleus, which results in expression of osteogenic genes such as Runt-related transcription factor 2 (RUNX2), collagen type 1, osteopontin (OPN), osteocalcin (OCN), and alkaline phosphatase (ALP) [2][8]. Also, the differentiation and proliferation of MSCs may be enhanced by physical factors like light-emitting diode (LED), low-level laser therapy or [9][10] photobiomodulation (PBM) therapy, and ultraviolet irradiation [11]. In recent years, there has been increasing attention on the improving effects of PBM on the osteogenic differentiation and proliferation of MSCs. [3]. PBM is a non-thermal procedure that uses LED light and low-level lasers to stimulate photo-sensitive receptors, such as intracellular water and cytochrome C oxidase triggering photochemical reactions in different biological pathways. [12]. Several studies investigated the elements that cause dental stem cell differentiation and bone regeneration. [13][14]. However, to date, only one study has evaluated the combined effects of PBM with 808 nm laser and vitamin D, as an anabolic factor, on osteogenic differentiation of HPDLSCs. [3]. However, the results of the aforementioned study are not conclusive for decision-making and therapeutic applications. More comprehensive information is required regarding the most efficient wavelength and intensity of laser to be applied in combination with vitamin D to achieve the highest level of osteogenic differentiation. This study aimed to assess the effects of PBM with 660 nm light (red light laser) with 3 and 5 J/cm2 density of energy [3][14] along with vitamin D (10-7 molar concentration) [3] on osteoblastic differentiation of HPDLSCs.

## Materials and Methods

**Table:** Table2. Measures of central dispersion for cell viability at 24 hours after the last laser irradiation cycle in the six groups

Group	Intervention	Sample size*	Mean	Std. deviation	Maximum	Minimum
1	OM(Control)	4	100.17	1.36	101.59	98.33
2	VD	4	106.05	0.78	106.98	105.12
3	660 nm, 10 s	5	105.95	2.09	107.91	103.25
4	VD + 660 nm, 10 s	4	109.48	4.70	116.04	104.88
5	660 nm, 17 s	5	120.79	6.26	127.67	114.88
6	VD + 660 nm, 17 s	5	131.02	3.06	135.11	127.91

^*^ One unit of cells in each of the groups 1, 2, and 4 was lost during the experiment, and their data could not be used in statistical analysis. OM: Osteogenic medium; VD: Vitamin D

**Table:** Table3. Pairwise comparisons of cell viability in the six groups at 24 hours

**I Group**	**J Group**	**Mean difference **	**P value**		**95% CI**
			**Lower bound **	**Upper bound **	
OM (Control) (1)	VD (2)	5.87	0.2	2.28-	14.02
OM (Control) (1)	660 nm, 10 s (3)	5.78	0.2	1.96-	13.51
OM (Control) (1)	VD+ 660 nm, 10 s (4)	9.30	0.02	1.15	17.46
OM (Control) (1)	660 nm, 17 s (5)	20.62	0.000	12.88	28.35
OM (Control) (1)	VD+ 660 nm, 17 s (6)	30.84	0.000	23.11	38.58
VD (2)	660 nm, 10 s (3)	0.09	1.00	7.64-	7.82
VD (2)	VD+ 660 nm, 10 s (4)	3.43	0.07	4.72-	11.58
VD (2)	660 nm, 17 s (5)	14.74	0.000	7.00	22.48
VD (2)	VD+ 660 nm, 17 s (6)	24.98	0.000	17.24	32.71
660 nm, 10 s (3)	VD+ 660 nm, 10 s (4)	3.52	0.07	4.21-	11.26
660 nm, 10 s (3)	660 nm, 17s (5)	14.83	0.000	7.54	22.13
660 nm, 10 s (3)	VD+ 660 nm, 17 s (6)	25.07	0.000	17.78	32.36
VD+ 660 nm, 10 s (4)	660 nm, 17 s (5)	11.31	0.002	3.58	19.04
VD+ 660 nm, 10 s (4)	VD+ 660 nm, 17s (6)	21.54	0.000	13.81	29.28
660 nm, 17 s (5)	VD+ 660 nm, 17s (6)	10.23	0.003	2.94	17.52

**OM:** Osteogenic medium; **VD:** Vitamin D; **CI:** Confidence interval

**Figure-1:**
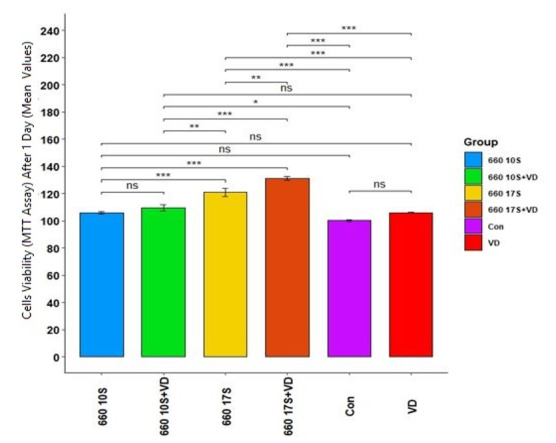
Figure 1. Cell viability as measured by the MTT assay at 24 hours post-irradiation. Con: Control group in culture medium, VD: cells treated with vitamin D in cell culture medium. Ns: not significant; *large difference from control (P<0.05), **large diference from control (P<0.01), ***large difference from control (P<0.001).

This experimental study was conducted in vitro on HPDLSCs obtained from the Dental Research Center of Tehran University of Medical Sciences. Human PDL cells were stained for STRO-1 (STRO: mesenchyme, 1: first isolated monoclonal antibody to identify mesenchymal stem cells), FACS (Fluorescence-activated cell sorting) sorted and expanded in culture. Human bone marrow SC (BMSC) served as a positive control This study method had been approved by the ethics committee of the Faculty of Dentistry, Shahed University, Tehran, Iran (ethical code: IR.SHAHED.REC.1401.054).

Size of Sample

Size of the sample in this study was calculated separately for each dependent variable, namely cell viability, gene expression, and calcification rate, using G-Power version 3.1.9.7 software (Erdfelder, Faul, & Buchner, 1996), assuming alpha=0.05, and study power of 0.80. Accordingly, the minimum sample size required for assessing the viability of cells by the methyl thiazolyl tetrazolium (MTT) assay was calculated to be 5 units of stem cells in each group (a total of 60 units for assessment of six groups at two different time points). The minimum sample size to assess these four gene expressions was calculated to be 3 units in each group (a total of 72). The minimum sample size for the assessment of calcification was calculated to be 10 units (a total of 180).

Study Groups

Six groups were evaluated as follows:

Group 0: Culture medium with no stem cell or intervention (this group only underwent Alizarin red staining).

Group 1 (control): HPDLSCs in the osteogenic medium with no intervention

Group 2: HPDLSCs in the osteogenic medium containing 10-7 molar vitamin D

Group 3 (660 nm, 10 s): HPDLSCs in the osteogenic medium were subjected to 660 nm exposure with 3 J/cm2 for 10 seconds.

Group 4 (VD + 660 nm, 10 s): HPDLSCs in the osteogenic medium containing 10-7 molar vitamin D were subjected to 660 nm laser irradiation with 3 J/cm2 density of energy for 10 seconds.

Group 5 (660 nm, 17 s): HPDLSCs in the osteogenic medium were subjected to 660 nm laser exposure with 5 J/cm2 energy density for 17 seconds.

Group 6 (VD + 660 nm, 17 s): HPDLSCs in the osteogenic medium containing 10-7 molar vitamin D were subjected to 660 nm laser exposure with 5 J/cm2 for 17 seconds.

Cell Culture and Intervention

HPDLSCs were cultured by the Dulbecco’s modified Eagle’s medium (DMEM; Cegrogen, Germany) with 10% fetal bovine serum (Gibco, UK), penicillin-streptomycin (idehZist) and L-glutamine (Cegrogen, Germany) at 37°C, 5% CO2, and 95% moisture. Third-fourth passage cells were seeded in a 96-well plate such that each well contained 10 x 106 cells, and incubated for 24 hours. Groups 2, 4, and 6 were exposed to 10-7 molar vitamin D for 48 hours. Vitamin D3 [1,25(OH)2D] was added to the culture medium in groups 2, 4, and 6. After 48 hours, groups 3 and 4 were subjected to laser exposure with 3 J/cm2 while groups 5 and 6 recieved 5 J/cm2 exposure. Low level 660 nm laser (konf Klas-DX; Konftec Corporation, Taiwan) with 150 mW power and an 8 mm2 hand-piece was irradiated in the continuous-wave mode to 0.5 cm2 spot size in the contact mode. Irradiation was performed in a completely dark room. The laser groups (3, 4, 5, and 6) were cultured in a separate container from the beginning to prevent light exposure. After seeding the cells received the initial laser exposure, and the second irradiation was performed 24 hours later.

Cell Viability Assessment (MTT Assay)

At day one (24h) and three (72h) after the laser exposure in groups 3-6, cell viability was assessed in all groups by the MTT assay (TACS, Trevigen, USA) [15]. For this purpose, centrifugation was performed, the supernatant was disposed, and each specimen received 50 µL of the MTT solution. Then the specimens were Incubated at 37°C and 5% CO2 for 3-4 hours. At the end, the MTT solution was discarded, and 60 µL of dimethyl sulfoxide was added to each specimen. The optical density of the wells was read at 570 nm wavelength by ELISA Reader. Cell viability was reported as the percentage of live cells compared to the control group (group 1) (100% viability) at each time point.

Assessment of Gene Expression by Reverse Transcription-polymerase Chain Reaction (RT-PCR)

To assess the expression of osteoblastic genes, PCR was carried out on specimens 14 days after the last laser irradiation (in groups 3-6). For this purpose, RNA of OCN, OPN, RUNX2, and ALP [15] was isolated by using phenol-chloroform and RNX Plus kit (Cinagen, Iran) according to the manufacturer's guide. The quality of the extracted RNA was evaluated by measuring the optical density at 260 nm wavelength and considering 280/260 nm reference wavelengths. Next, cDNA was synthesized from 1 µg of RNA using the Add Bio kit. Quantitative real-time RT-PCR was conducted by SYBR Green PCR Master Mix (SMO BIO, China) using LightCycler® 96 (Roche, Basel, Switzerland) system. The nucleotide sequence of the PCR primers is shown in Table-1. The level of expression of each gene relative to the expression of the GAPDH housekeeping gene [16] was evaluated, and the results were analyzed using the 2 −ΔCT formula.

Assessment of Calcification

To induce osteoblastic differentiation in groups 1-6, the cells were allocated in a 24-well plate containing DMEM with 5% fetal bovine serum, 10 nM dexamethasone, 50 µg/mL ascorbic acid (Sigma Aldrich, Germany), and 10 mM b-glycerophosphate at a density of 80,000 cells/well. The culture medium was refreshed every 2 days. The cells treated with DMEM containing 10% fetal bovine serum and penicillin-streptomycin were considered the negative control group (group 0). Groups 2, 4, and 6 were exposed to 10-7 molar vitamin D for 48 hours. After 48 hours, groups 3 and 4 received 3 J/cm2 exposure, and groups 5 and 6 received 5 J/cm2 laser irradiation. Alizarin Red staining of the cells was performed on day 21 after the last laser irradiation (in groups 3-6) in all groups. To identify calcifications, cultured cells were rinsed with phosphate-buffered saline twice, and fixed with 10% formalin (Roth, Germany) for 10 minutes. Next, they were hydrated with 1 mL of distilled water for 5 minutes and stained with 200 µL of 1% Alizarin Red S stain (Sigma, Germany) with a pH of 4. Next, the Red S solution was eliminated, and the specimens were rinsed again with phosphate-buffered saline for 15 minutes. The intensity of staining was evaluated by photography of the culture plates under similar environmental and lighting conditions. The photographs were analyzed by Image J software (National Institutes of Health, Bethesda, Maryland, USA) in comparison with group 0.

Statistical Analysis

Data has been analyzed by R version 4.2.1 software (R Core Team, Austria) and its dplyr, static, and PMCMR plus packages. Considering the normal distribution of cell viability data as confirmed by the Shapiro-Wilk test (P>0.05) and homogeneity of variances as confirmed by the Levene’s test (P>0.05), one-way ANOVA was applied to analyze the effects of vitamin D and laser irradiation (3 and 5 J/cm2) on cell viability. The Tukey test performed pairwise comparisons of cell viability. The effects of vitamin D and laser irradiation (3 and 5 J/cm2) on RUNX2, ALP, OPN, and OCN gene expressions were analyzed by the Kruskal-Wallis test (due to non-normal distribution of data shown by the Shapiro-Wilk test), which was followed by pairwise comparisons with the Multiple Comparisons of Mean Rank Sums (Conover’s test) with Bonferroni adjustment. The effects of vitamin D and laser irradiation (3 and 5 J/cm2) on calcification were assessed by the Welch test due to the normal distribution of data as confirmed by the Shapiro-Wilk test and non-homogeneity of variances as shown by the Levene’s test. Pairwise comparisons of the groups regarding calcification were performed by the Games-Howell test. The level of statistical significance was set at 0.05.

## Results

**Table:** Table4. Measures of central dispersion for cell viability 72 hours after the last beam exposure cycle for all six groups

Group	Intervention	Sample size*	Mean	Std. deviation	Maximum	Minimum
1	OM(Control)	4	99.53	1.26	101.17	98.54	
2	VD	4	102.54	1.11	104.18	101.74	
3	660nm 10s	5	99.63	3.40	102.87	95.91	
4	VD 660 nm 10s	5	104.07	1.89	105.88	102.11	
5	660nm 17s	5	111.51	2.36	114.66	109.00	
6	VD 660nm 17s	5	113.74	1.98	116.78	112.27	

^*^ One unit of cells in each of groups 1 and 2 was lost during the experiment, and their data could not be used in statistical analysis. **OM:** Osteogenic medium; **VD:** Vitamin D

**Table:** Table5. Pairwise comparisons of cell viability in the six groups at 72 hours

(I) Group	(J) Group	Mean difference	P value		95% CI
			Lower bound	Upper bound	
OM(Control) (1)	VD(2)	3.00	0.4	1.85-	7.87
OM(Control) (1)	660nm, 10s(3)	0.10-	1.00	4.71-	4.51
OM(Control) (1)	VD+ 660nm, 10s(4)	4.54	0.05	0.07-	9.15
OM(Control) (1)	660nm, 17s(5)	11.99	0.000	7.38	16.60
OM(Control) (1)	VD+ 660nm, 17s(6)	14.21	0.000	9.60	18.81
VD(2)	660nm, 10s(3)	2.90	0.3	1.70-	7.51
VD(2)	VD+ 660nm, 10s(4)	1.53	0.9	3.08-	6.14
VD(2)	660nm, 17s(5)	8.98	0.000	4.37	13.59
VD(2)	VD+ 660nm, 17s(6)	11.19	0.000	6.59	15.81
660nm, 10s(3)	VD+ 660nm, 10s(4)	4.44	0.04	0.09	8.78
660nm, 10s(3)	660nm, 17s(5)	11.88	0.000	7.54	16.23
660nm, 10s(3)	VD+ 660nm, 17s(6)	14.10	0.000	9.76	18.45
VD+ 660nm, 10s(4)	660nm, 17s(5)	7.45	0.000	3.100	11.79
VD+ 660nm, 10s(4)	VD+ 660nm, 17s(6)	9.67	0.000	5.32	14.01
660nm, 17s(5)	VD+ 660nm, 17s(6)	2.21	0.6	2.13-	6.56

**OM:** Osteogenic medium; **VD:** Vitamin D

Cell Viability

24 hours: Table-2 presents the measures of central dispersion for cell viability after the final beam exposure at the 24th hour (in groups 3-6) in the six groups. As shown, the highest and the lowest mean cell viability was noted in group 6 and group 3 (without considering the control group), respectively. the six groups had a major difference (P=0.000). In other words, PBM with 660 nm laser and vitamin D had a major impact on the viability of the specimens after 24 hours. The effect size was found to be 0.91, indicating the high effect of the intervention. Table-3 (Pairwise comparisons) showed a big distinctness between all groups (P<0.05) but between groups 1 (control) and 3, control and 2, 2 and 3, 2 and 4, and 3 and 4 (P>0.05, Figure-1).

**Figure-2:**
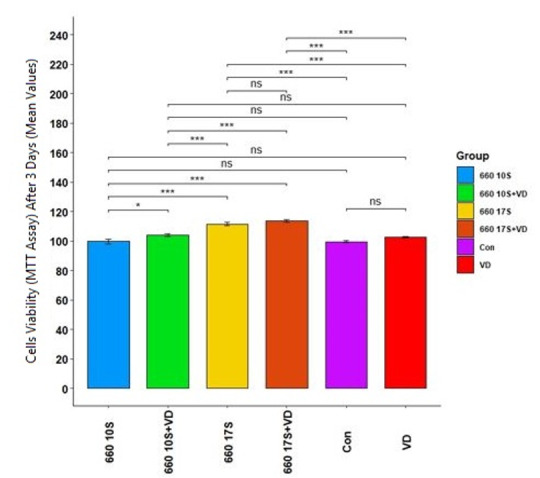
Figure 2. Cell viability as measured by the MTT assay at 24 hours post-irradiation. Con: Control group in the culture medium, VD: cells treated with vitamin D in cell culture medium. Ns: not significant; *large difference from control (P<0.05), **large difference from control (P<0.01), ***large difference from control (P<0.001).

72 hours: Table-4 presents the measures of central dispersion for cell viability at day three after the final beam exposure cycle (in groups 3-6) in the six groups. As shown, the highest and the lowest mean cell viability was noted in group 6 and group 3, respectively (without considering the control group). A notable contrast had been seen in the cell viability of the six groups (P=0.000). In other words, PBM with 660 nm laser and vitamin D did affect cell viability after 72 hours. The effect size was found to be 0.89, indicating the high effect of the intervention. Table-5 (Pairwise comparisons) demonstrated the huge contrast in all groups (P<0.05) except between groups 1 (control) and 2, control and 3, control and 4, 2 and 4, 2 and 3, and 5 and 6 (P>0.05, Figure-2).

Osteogenic Gene Expression

Table-6 demonstrates the evaluation of central dispersion for the expression of genes in the six groups. Group 4 showed the highest expression of RUNX2 and ALP, and was considered as the reference group (expression of 1). Thus, expression of genes in other groups was compared with this group. Group 6 was considered as the reference group (expression of 1) for OPN and OCN genes.

RUNX2: The highest and the lowest expression of the RUNX2 gene was noted in groups 4 and 1, respectively. A wide diversity had been retrieved in the expression of RUNX2 among the six groups (P=0.005), indicating the significant PBM effect by 660 nm beam and vitamin D on the expression of RUNX2. The eta 2 was found to be 0.94, indicating the high effect of PBM and vitamin D on RUNX2 expression. A large diversity had been revealed in pairwise comparisons between all groups (P=0.000) except comparing groups 4 and 6 (P=0.06). In other words, only groups 4 and 6 had no significant difference with each other, and all other groups showed lower expression of RUNX2 than the reference group, indicating that PBM with 660 nm laser along with vitamin D significantly affected the expression of RUNX2. In contrast, the duration of irradiation didn’t impact the RUNX2 expression notably.

ALP: The highest and the lowest expression of the ALP gene was noted in groups 4 and 5, respectively. Large diversity had been found in the expression of ALP among the six groups (P=0.007), indicating the significant effect of PBM with 660 nm laser and vitamin D on the expression of ALP. The eta 2 was found to be 0.89, indicating the high effect of PBM and vitamin D on ALP expression. Large dissimilarity was found by comparing pairwise of all groups (P=0.000 for all, except P=0.005 for the difference between groups 3 and 4) except between groups 4 and 6 (P=0.3) which had no significant difference with each other; all other groups showed lower expression of ALP than the reference group, indicating that PBM with 660 nm laser along with vitamin D significantly affected the expression of ALP. In contrast, the duration of irradiation did not affect the ALP expression significantly.

OPN: The highest and the lowest expression of the OPN gene was noted in groups 6 and 1, respectively. A noteworthy change had been seen in the expression of OPN among the groups (P=0.007), indicating the significance of 660 nm laser and vitamin D effect on the expression of OPN. The eta 2 was found to be 0.90, indicating the high effect of PBM and vitamin D on OPN expression.

Significant differences were observed among all groups in Pairwise comparisons (P=0.000 for all except P=0.01 for the difference between the second and sixth groups), except between groups 6 (reference) and 4 (P=0.06) which had no significant difference with each other. Other groups showed lower expression of OPN than the reference group, indicating that PBM with 660 nm laser along with vitamin D significantly affected the expression of OPN while duration of irradiation could not affect expression of OPN significantly.

OCN: The highest and the lowest expression of the OCN gene was noted in groups 6 and 1, respectively.

A noteworthy change had been seen in the OCN expression among the six groups (P=0.005), indicating the significant role of 660 nm irradiation and vitamin D on the expression of OCN. The eta 2 was found to be 0.97, indicating the high effect of PBM and vitamin D on OPN expression. Significant differences were observed among all groups in Pairwise comparisons (P=0.000 for all except P=0.008 for the difference between groups 4 and 6). In other words, all groups showed lower expression of OCN than the reference group, indicating the significant role of 660 nm irradiation and vitamin D and also the duration of irradiation on the expression of OCN.

Calcification

**Figure-3:**
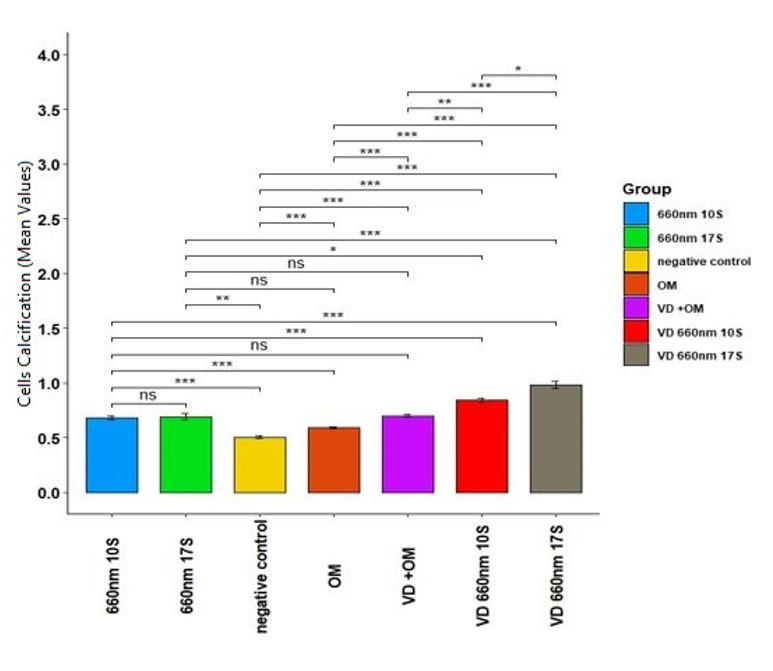
Figure 3. Mean intensity of calcium deposition by using Alizarin red staining. OM: osteogenic medium; VD: Vitamin D. *large difference from control (P<0.05), **large difference from control (P<0.01), ***large difference from control (P<0.001).

Table-7 shows the amounts of central dispersion for calcification in the groups. The maximum and the minimum mean rates of calcification were noted in groups 6 and 0 (control), respectively. A large difference had been seen in the rate of calcification among the groups (P=0.000), indicating the significant effect of PBM with vitamin D on calcification. The Eta2 for the effect size was found to be 0.85, indicating the large impact of 660 nm irradiation and vitamin D on calcification. Pairwise comparisons (Table-8) showed huge differences in all groups (P<0.05) except between groups 1 and 5, 2 and 3, and 3 and 5 (Figure-3).

## Discussion

**Table:** Table6. Measures of central dispersion for the expression of genes in the six groups (n=3)

Gene	Group number	Group	Mean	Std. deviation	Maximum	Minimum
	1	OM	0.49	0.03	0.53	0.47
	2	VD	0.76	0.06	0.83	0.70
RUNX2	3	660nm, 10s	0.73	0.03	0.76	0.70
	4	VD+ 660 nm, 10s	1.00	0.00	1.00	1.00
	5	660nm, 17s	0.57	0.04	0.61	0.54
	6	VD+ 660nm, 17s	0.88	0.05	0.94	0.84
	1	OM	0.49	0.005	0.50	0.48
	2	VD	0.49	0.005	0.50	0.48
ALP	3	660nm, 10s	0.74	0	0.74	0.74
	4	VD+ 660 nm, 10s	1.00	0	1.00	1.00
	5	660nm, 17s	0.48	0.007	0.49	0.47
	6	VD+ 660nm, 17s	0.86	0.03	0.89	0.83
	1	OM	0.38	0.005	0.39	0.38
	2	VD	0.78	0.1	0.79	0.76
OPN	3	660nm, 10s	0.46	0.006	0.47	0.46
	4	VD+ 660nm, 10s	0.78	0.01	0.79	0.77
	5	660nm, 17s	0.39	0.005	0.39	0.38
	6	VD+ 660 nm, 17s	1.00	0.00	1.00	1.00
	1	OM	0.32	0.008	0.33	0.31
	2	VD	0.61	0.02	0.63	0.59
OCN	3	660nm, 10s	0.45	0.01	0.46	0.44
	4	VD+ 660nm, 10s	0.77	0.02	0.79	0.75
	5	660nm, 17s	0.38	0.01	0.39	0.37
	6	VD+ 660 nm, 17s	1.00	0.00	1.00	1.00

**OM:** Osteogenic medium; **VD:** Vitamin D; OCN; OPN; GAPDH; ALP; RUNX2

**Table:** Table7. Measures of central dispersion for calcification in the groups

Group	Intervention	Sample size*	Mean	Std. deviation	Maximum	Minimum
0	Negative(No cell)-control	10	0.51	0.08	0.71	0.43	
1	OM	9	0.59	0.02	0.63	0.55	
2	VD	9	0.70	0.05	0.75	0.64	
3	660nm, 10s	10	0.68	0.04	0.74	0.61	
4	VD+ 660 nm, 10s	8	0.84	0.06	0.92	0.75	
5	660nm, 17s	10	0.69	0.09	0.84	0.58	
6	VD+ 660nm, 17s	9	0.98	0.09	1.01	0.83	

^*^ One unit of cells in each of the groups 1, 2, and 6, and two units of cells in group 4 were lost during the experiment, and their data could not be used in statistical analysis. OM: Osteogenic medium; VD: Vitamin D

**Table:** Table8. Calcification: Pairwise comparing of all groups (Games-Howell test)

(I) Group	(J) Group	Mean difference	P value		95% CI
			Lower bound	Upper bound	
Negative(No cell)-control (0)	OM(1)	0.09	0.000	0.04	0.14
Negative(No cell)-control (0)	VD (2)	0.19	0.000	0.13	0.26
Negative(No cell)-control (0)	660nm, 10s(3)	0.18	0.000	0.11	0.24
Negative(No cell)-control (0)	VD+ 660nm, 10s(4)	0.34	0.000	0.25	0.42
Negative(No cell)-control (0)	660nm, 17s(5)	0.19	0.002	0.07	0.30
Negative(No cell)-control (0)	VD+ 660nm, 17s(6)	0.48	0.000	0.35	0.60
OM(1)	VD (2)	0.11	0.000	0.05	0.16
OM(1)	660nm, 10s(3)	0.09	0.000	0.03	0.14
OM(1)	VD+ 660nm, 10s(4)	0.25	0.000	0.16	0.33
OM(1)	660nm, 17s(5)	0.09	0.1	0.18	0.22
OM(1)	VD+ 660nm, 17s(6)	0.39	0.000	0.27	0.51
VD (2)	660nm, 10s(3)	0.02	0.09	0.05-	0.08
VD (2)	VD+ 660nm, 10s(4)	0.14	0.002	0.05	0.23
VD (2)	660nm, 17s(5)	0.008	1.00	0.11-	0.13
VD (2)	VD+ 660nm, 17s(6)	0.28	0.000	0.16	0.40
660nm, 10s(3)	VD+ 660nm, 10s(4)	0.16	0.000	0.07	0.25
660nm, 10s(3)	660nm, 17s(5)	0.01	1.00	0.11-	0.13
660nm, 10s(3)	VD+ 660nm, 17s(6)	0.30	0.000	0.18	0.42
VD+ 660nm, 10s(4)	660nm, 17s(5)	0.15	0.01	0.02	0.28
VD+ 660nm, 10s(4)	VD+ 660nm, 17s(6)	0.14	0.03	0.000	0.27
660nm, 17s(5)	VD+ 660nm, 17s(6)	0.29	0.000	0.14	0.44

**OM:** Osteogenic medium; **VD:** Vitamin D

We conducted this study to examine the effect of PBM in 660 nm laser (red light laser) by 3 and 5 J/cm2 energy density [3][14] along with vitamin D (10-7 molar concentration) [3] on osteoblastic differentiation of HPDLSCs. The results concluded an increase in viability of cells in all groups after 24 hours. Application of a 660 nm laser in 5 J/cm2 plus vitamin D was the most efficient modality to increase cell viability after 24 hours. Cell viability further increased in all groups at 72 hours, except in group 3.

Application of a 660 nm laser with 5 J/cm2 with/without vitamin D was the most efficient modality to increase cell viability after 72 hours. Regarding gene expression, the results showed that the synergistic effect of vitamin D and laser (both 3 and 5 J/cm2) was effective for up-regulation of RUNX2, and the duration of laser irradiation played no significant role in this regard. The same results were obtained for ALP and OPN gene expressions. The synergistic effect of vitamin D and 5 J/cm2 laser was the most efficient for the upregulation of OCN, and the duration of laser irradiation also played a role in this regard. Regarding calcification, our results demonstrated that the application of 660 nm laser with 3 and 5 J/cm2 energy density increased calcification, and the synergistic effect of vitamin D and 5 J/cm2 laser irradiation was greater than the effect of each modality alone. The duration of laser irradiation also played a role in this regard.

Abdelgawad et al. [3] assessed the effect of photo biomodulation and vitamin D on osteoblastic differentiation of HPDLSCs and new bone formation by assessing activity of enzymes and expression of genes. They used 10-7 M vitamin D and applied diode laser with 808 nm beam with 1 and 2 J/cm2 energy density alone and together. They indicated that all interventions increased cell viability, osteogenic gene expression, and calcification. PBM was more effective than vitamin D, and the combined effect of vitamin D and irradiation (both 1 and 2 J/cm2) had the highest efficacy for cell viability, proliferation, and differentiation through the expression of osteogenic genes. These outputs were similar with the present results although using a different wavelength and energy density of the laser. Pinheiro and Bueno [17] used a 660 nm irradiation for the multiplication or differentiation of MSCs and reported that an irradiation by 660 nm with 2 J/cm2 energy density had the highest efficacy for this purpose and increased calcification. Those finding were in concordance with recent results despite using different energy densities. Kreisler et al. [18] assessed the effect of low-level 809 nm GaAlAs laser with 10 mW power and 1.96-7.84 J/cm2 energy density in continuous-wave mode on the proliferation of PDL fibroblasts. They reported significantly higher cell proliferation in the irradiated groups than in the control group even 72 hours after the irradiation. present study revealed similar results although the present study also assessed the synergistic effect of vitamin D and laser. In total, Abdelgawad al. [3] reported the optimal efficacy of low-level 808 nm laser with up to 2 J/cm2 energy density, and Choi et al, [19] and Wu et al. [20] discussed that the stimulating effect of 808 nm laser is present in energy densities up to 4 J/cm2.

According to Bouvet-Bouvet-Gerbettaz et al, [21] energy densities higher than 4 J/cm2 would harm the viability of osteoblasts. However, Kreisler et al. [18] used an 808 nm laser with up to 8 J/cm2 energy density and reported its positive effect on cell viability. In our study, 660 nm beam was used at 3 and 5 J/cm2 densities of energy and the results showed that a 660 nm irradiation at 5 J/cm2 with or without vitamin D was the most efficient modality to increase cell viability at 72 hours.

On the other hand, RUNX2 is the main gene known to regulate the MSC osteogenic differentiation [3].

This study revealed that the synergistic effect of vitamin D and laser with 3 and 5 J/cm2 energy densities was suitable for upregulation of RUNX2, and duration of irradiation had no prominent role in this respect. The upregulation of RUNX2 by laser irradiation was in agreement with the results of Peng et al, [22] who reported that irradiation of 620 nm red LED with 2 J/cm2 energy density up-regulated RUNX2 in bone marrow stem cells. Similarly, Abdelgawad et al. [3] demonstrated gene expression increases after adding vitamin D with irradiation of 808 nm laser, and increasing the energy density from 1 to two J/cm2 gene expression further increased, similar to the present findings. Ji et al. [23] found that the application of vitamin D (similar to group 2 of the present study) up-regulated RUNX and OCN genes. Also, Wang et al. [24] showed osteoblastic differentiation of periosteal cells following the application of vitamin D with the same concentration used in the present study (similar to the second group of our study). However, our study also indicated the synergistic effect of vitamin D (10-7 molar) and 660 nm beam at 5 J/cm2 on calcification, which was greater than the effect of each modality alone. Abdelgawad et al. [3] Also found the maximum rate of calcification in the group subjected to 10-7 molar vitamin D and 880 nm irradiation with 2 J/cm2.

## Conclusion

Our results showed that combined use of vitamin D3 and irradiation of 660 nm laser with 3 J/cm2 and particularly 5 J/cm2 energy density increased the viability of HPDLSCs and enhanced their osteoblastic differentiation.

## Conflict of Interest

The authors certify that they have NO affiliations with or involvement in any organization or entity with any financial or non-financial interest in the subject matter or materials discussed in this manuscript.
